# West Nile Virus as a Trigger for Acute Inflammatory Demyelinating Polyneuropathy: Exploring Intravenous Immunoglobulin (IVIG) Efficacy and Disease Variability

**DOI:** 10.7759/cureus.100439

**Published:** 2025-12-30

**Authors:** Matthew D Howard, Justin Baman, Ramsha Bhutta

**Affiliations:** 1 Internal Medicine, St. Francis Hospital, Columbus, USA; 2 Psychiatry, St. Francis Hospital, Columbus, USA; 3 Neurology, St. Francis Hospital, Columbus, USA

**Keywords:** acute inflammatory demyelinating polyneuropathy (aidp), demyelinating polyneuropathy, guillain-barré syndrome, immune-mediated neuropathy, intravenous immunoglobulin therapy, neuroinfectious disease, post-infectious demyelination, west nile virus infection

## Abstract

West Nile virus (WNV) is a rare trigger of acute inflammatory demyelinating polyneuropathy (AIDP), more commonly associated with meningoencephalitis. This case highlights an atypical post-infectious presentation of WNV-associated AIDP following recent viral illness and explores the associated diagnostic and therapeutic challenges. A 36-year-old male patient with heavy alcohol use, chronic tobacco exposure, and a recent upper respiratory infection developed rapidly progressive bilateral weakness, numbness, and paresthesias over several days, ultimately becoming unable to walk. Examination revealed areflexia, distal-predominant sensory loss, ataxia, and cerebellar tremor. Cerebrospinal fluid analysis showed albuminocytologic dissociation, and serologic testing was positive for WNV immunoglobulin M (IgM) and immunoglobulin G (IgG), suggesting recent or ongoing infection. Electrodiagnostic studies demonstrated a mixed demyelinating and axonal polyneuropathy with secondary axonal loss. Brain MRI revealed a small, nonspecific focus of possible demyelination, while spine MRI showed no nerve root enhancement. The patient was treated with a standard five-day course of intravenous immunoglobulin (IVIG) with respiratory monitoring and supportive care, resulting in gradual strength improvement and eventual restoration of functional mobility. This case emphasizes the diagnostic complexity of distinguishing WNV-associated AIDP from other neuroinvasive or immune-mediated neurologic conditions and underscores the importance of early recognition and timely immunotherapy. In addition, the patient’s significant alcohol and tobacco exposure highlights the potential influence of comorbid substance use on immune function and recovery trajectory. Although our patient experienced favorable improvement with standard therapy, prior reports suggest potential variability in IVIG responsiveness among WNV-associated neuropathies, representing an important area for further investigation.

## Introduction

West Nile virus (WNV) is an established neuroinvasive pathogen most commonly associated with meningoencephalitis and acute flaccid paralysis; however, it has also been implicated, though less frequently, as a trigger for immune-mediated peripheral nervous system disorders such as acute inflammatory demyelinating polyneuropathy (AIDP), a variant of Guillain-Barré syndrome (GBS) [1]. Atypical presentations of AIDP following WNV infection highlight the need for greater clarity regarding the underlying immune mechanisms. While neuroinvasive infection may reflect impaired innate or adaptive immune defense, emerging evidence suggests that dysregulated T-cell responses also contribute to neuropathogenesis. In particular, atypically polarized WNV-specific T cells have been observed in patients with neuroinvasive disease compared with asymptomatic individuals [2].

According to the American Society of Transplantation Infectious Diseases Community of Practice, WNV-specific intravenous immunoglobulin (IVIG) has been utilized in various WNV-associated neuropathies; however, reported outcomes have been variable and may depend on factors such as timing of administration and the presence of neutralizing antibodies [3]. Despite these observations, there remains limited guidance on distinguishing post-infectious AIDP from primary neuroinvasive WNV presentations, as well as limited data on predictors of treatment responsiveness in this setting [4].

Comorbid substance exposure may further influence immune function and disease course. Although supporting literature remains limited, cannabis and nicotine exposure have been associated with altered immune regulation and cytokine signaling, which may plausibly affect vulnerability to infection or recovery trajectories in immune-mediated neuropathies [5,6].

## Case presentation

A 36-year-old male patient with alcohol use disorder, consuming approximately eight drinks daily and a 20-pack-year smoking history, presented with rapidly progressive bilateral extremity weakness, numbness, and paresthesias over three days. One week prior, he experienced a self-limited viral upper respiratory illness with cough that resolved without treatment. His neurologic symptoms began with left arm paresthesias and weakness, gradually spreading to the right arm and both lower extremities, ultimately resulting in the inability to ambulate. He reported mild back pain but denied bowel or bladder incontinence, saddle anesthesia, dysphagia, dyspnea, visual changes, or constitutional symptoms. He was not taking any medications, had no known allergies, and had no prior surgeries or relevant family history. There was no recent travel, tick exposure, or outdoor exposure risk. The patient reported regular alcohol use and chronic tobacco smoking. He also acknowledged recent cannabis use, which was consistent with a positive urine cannabinoid screen.

On arrival, vital signs were stable. Cranial nerves were intact. Motor examination demonstrated symmetric weakness graded 3/5 in the upper extremities and 2/5 in the lower extremities. Sensory examination revealed decreased pinprick and vibration sensation in all extremities, more pronounced distally, with associated paresthesias. Deep tendon reflexes were absent throughout. He demonstrated dysmetria on finger-to-nose testing and gait ataxia. These findings were felt to be most consistent with sensory ataxia, given the significant distal sensory loss, although a central contribution could not be completely excluded. There were no skin lesions suggestive of tick-borne disease, and straight-leg raise testing was negative.

Serum WNV immunoglobulin M (IgM) and immunoglobulin G (IgG) antibodies were positive and were obtained shortly after neurologic symptom onset, supporting recent infection as a potential immune trigger, although IgG positivity alone may reflect prior exposure. Lyme serology and fourth-generation HIV testing were negative, and the thiamine level was not obtained. Lumbar puncture demonstrated albuminocytologic dissociation with elevated protein and no pleocytosis, and CSF culture showed no growth. Further evaluation for both immune-mediated neuropathy and potential neuroinvasive etiologies included CSF WNV antibody testing, a CSF encephalitis panel, CSF Lyme Western blot, and CSF anti-GQ1b and GM1 antibodies; however, these send-out studies ultimately did not result.

**Figure 1:**
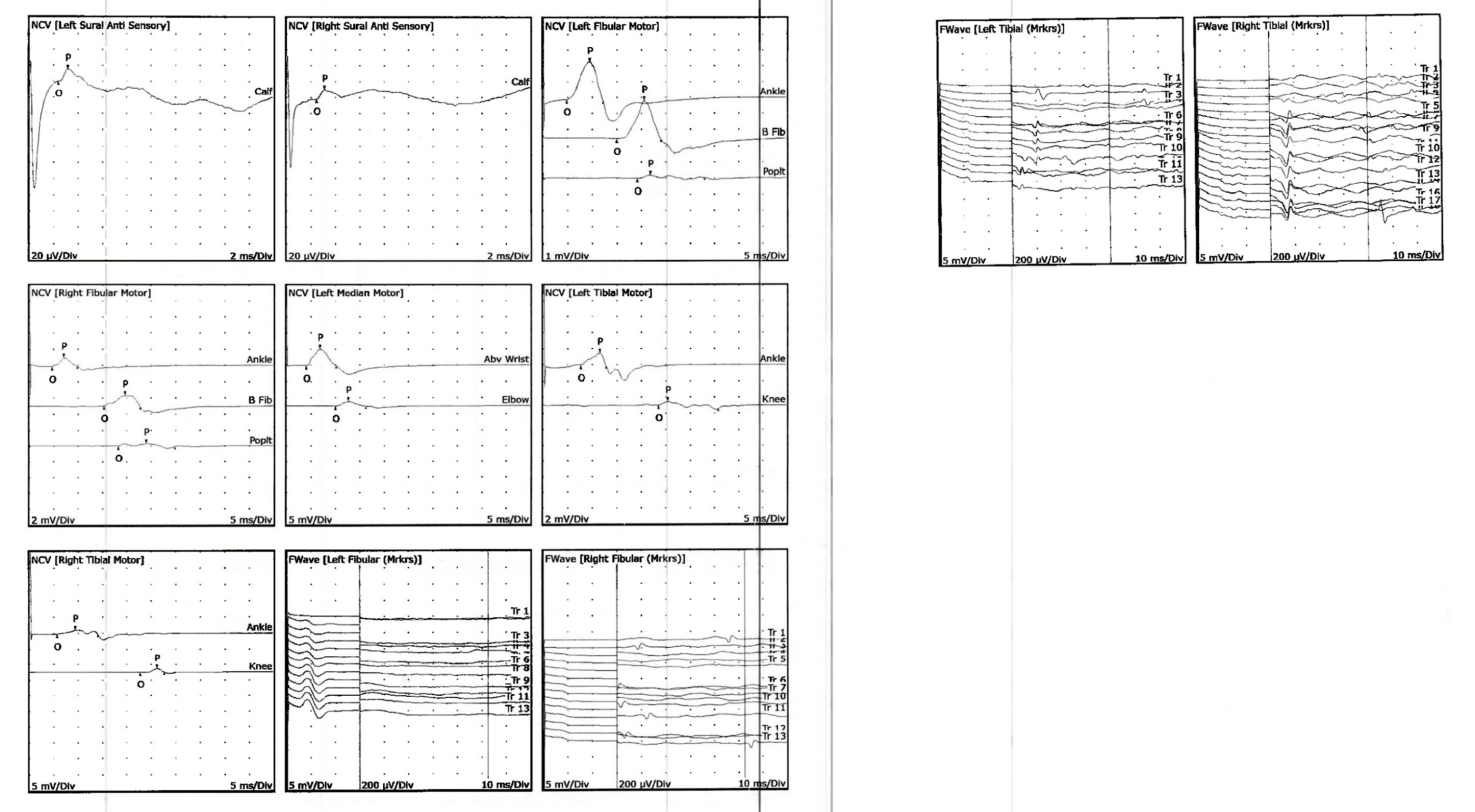
Nerve conduction studies demonstrating widespread, distal sensorimotor demyelinating polyneuropathy with secondary axonal loss

**Figure 2:**
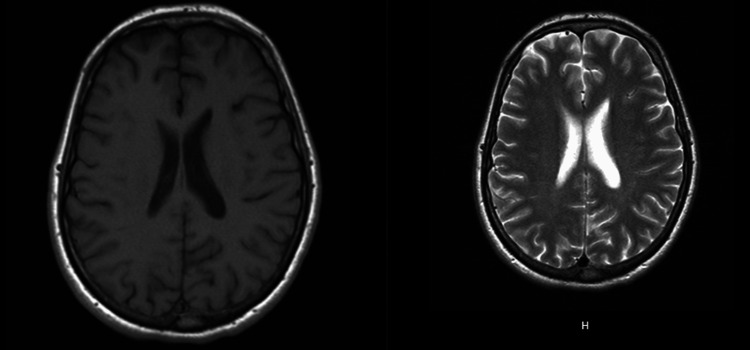
Axial T1-weighted image showing a subtle hypointense focus in the left parietal white matter Corresponding T2-weighted image demonstrating hyperintense signal in the same region. Together, these findings are suggestive of an area of focal demyelination

Electrodiagnostic testing demonstrated findings consistent with a widespread acquired demyelinating polyneuropathy with secondary axonal involvement. Nerve conduction studies revealed markedly reduced conduction velocities, prolonged distal latencies, reduced amplitudes, and clear conduction block across multiple motor nerves in both lower extremities and the left upper extremity. Sensory nerve responses were reduced in amplitude with prolonged distal peak latencies, supporting a sensorimotor process rather than a purely motor anterior horn cell disease. Late response abnormalities were notable, with prolonged fibular and tibial F-wave latencies bilaterally, indicating proximal demyelination and root-level involvement. Needle electromyography (EMG) demonstrated spontaneous activity in select right lower extremity muscles, suggesting superimposed localized axonal injury; however, the overall pattern favored a diffuse demyelinating polyradiculoneuropathy rather than acute motor axonal neuropathy or WNV-associated anterior horn cell disease. Additional axonal loss isolated to the right lower extremity suggested a possible superimposed radiculopathy (Figure 1). Brain MRI demonstrated a small left parietal white matter focus that appeared hypointense on T1-weighted imaging and hyperintense on T2-weighted imaging, consistent with a nonspecific white matter abnormality. There was no associated enhancement, mass effect, or diffusion restriction. Importantly, this finding did not demonstrate the radiographic characteristics typically associated with acute demyelinating disease or encephalitis and was considered incidental. MRI of the cervical, thoracic, and lumbar spine demonstrated no anterior horn involvement, cord lesions, or nerve root enhancement, which, when integrated with symmetric weakness, sensory involvement, and albuminocytologic dissociation, supported WNV-associated AIDP rather than WNV-associated acute flaccid myelitis (AFM) (Figure 2). Additionally, MRI of the cervical, thoracic, and lumbar spine with and without contrast showed no nerve root enhancement, cord signal abnormality, or evidence of anterior horn involvement.

Management and clinical progression

The patient was promptly initiated on IVIG therapy at 0.4 g/kg daily for five days and transferred to the step-down intensive care unit for close respiratory monitoring. Forced vital capacity and negative inspiratory force were monitored every six hours, in addition to frequent neurologic examinations. His respiratory parameters remained stable, with an initial negative inspiratory force of -70 cm H₂O and no signs of respiratory compromise. Daily physical and occupational therapy were initiated, and plasmapheresis was designated as a contingency plan should neurological deterioration occur. Given his history of heavy alcohol use, a Clinical Institute Withdrawal Assessment (CIWA) protocol was initiated along with a benzodiazepine taper and thiamine supplementation to reduce the risk of alcohol withdrawal and Wernicke’s encephalopathy.

Clinical improvement progressed gradually. By day 2 of IVIG therapy, he demonstrated mild improvement in strength, although paresthesias and ataxia persisted. There was no appreciable change on days 3 and 4. By day 5, he exhibited substantial recovery in strength with persistent but improving distal sensory loss and residual ataxia. With continued improvement and no concern for respiratory decline, he was transferred to the general medical floor. Over subsequent therapy sessions, gait steadily improved; initially requiring a rolling walker and gait belt, he progressed to independent ambulation with normalization of cadence. Gabapentin was initiated for neuropathic symptoms with good symptomatic relief. He was discharged home with outpatient neurology follow-up, continued physical therapy, and mild residual distal sensory symptoms, but no motor deficits or ataxia.

## Discussion

WNV and AIDP

WNV is an arbovirus capable of causing a broad clinical spectrum, from asymptomatic infection to severe neuroinvasive disease. AIDP is a recognized but rare immune-mediated complication of WNV infection, occurring in a subset of patients who develop a post-infectious peripheral demyelinating neuropathy rather than direct viral motor-neuron injury [4].

WNV-associated AIDP typically presents 1-8 weeks after the initial infection with rapidly progressive symmetric limb weakness, generalized areflexia, and variable sensory or autonomic involvement. This contrasts with WNV AFM, which is classically asymmetric, purely motor, and often accompanied by spinal cord or anterior horn abnormalities on MRI [7].

Serological testing helps support the diagnosis as WNV IgM indicates recent infection and may persist for months, while IgG alone may reflect prior exposure. Cross-reactivity with other flaviviruses is possible, so clinical correlation is essential. CSF IgM is more specific for neuroinvasive disease, though its absence does not exclude immune-mediated peripheral neuropathy [8]. CSF WNV IgM can provide supportive diagnostic value in suspected neuroinvasive infection; in our patient, these studies were obtained but ultimately did not result. However, the absence of pleocytosis, negative CSF cultures, lack of encephalitic features, and the combination of symmetric weakness with sensory involvement, albuminocytologic dissociation, nonenhancing MRI, and demyelinating electrodiagnostic findings favored a postinfectious immune-mediated polyradiculoneuropathy rather than direct neuroinvasive WNV disease. MRI is typically normal in WNV-associated AIDP because the disease primarily affects peripheral nerves. Neuroimaging is used mainly to exclude central etiologies or to detect features of encephalitis, meningitis, or myelitis, which more reliably demonstrate radiographic abnormalities [9]. Previous studies of WNV-associated AIDP demonstrate highly variable outcomes, ranging from rapid clinical improvement to minimal or no recovery despite IVIG treatment [10]. Previous reports describe variable outcomes in WNV-associated AIDP, although systematic treatment data are limited. Randomized evidence does not demonstrate the benefit of IVIG in neuroinvasive WNV infection, and current CDC guidance does not support its routine use. Importantly, distinguishing immune-mediated demyelinating polyradiculoneuropathy from direct viral anterior horn cell injury (WNV-associated AFM) is essential, as these conditions have distinct prognoses and characteristic electrodiagnostic profiles [10,11].

Diagnostic criteria for AIDP

AIDP is diagnosed through a combination of clinical, CSF, and electrodiagnostic criteria. Clinically, AIDP is characterized by rapidly progressive, symmetrical limb weakness evolving over hours to up to four weeks; areflexia or hyporeflexia; and a monophasic course with a diagnostic nadir reached within four weeks. Sensory symptoms such as paresthesias may be present but are less prominent than motor deficits. Exclusion of toxic, metabolic, infectious, or structural mimics is essential [12]. CSF analysis typically demonstrates albuminocytologic dissociation, elevated protein with normal or mildly increased cell count, as seen in our patient. Although emerging CSF biomarkers such as neurofilament light chain (NfL), neurofilament heavy chain (NfH), glial fibrillary acidic protein (GFAP), sphingomyelin, and brain-derived neurotrophic factor (BDNF) may enhance diagnostic precision in inflammatory demyelinating polyneuropathies, these assays were not obtained in our patient and remain primarily research-focused rather than routinely available in clinical practice [13].

Imaging and electrophysiological variability

MRI is not diagnostic for AIDP but may demonstrate gadolinium enhancement of the cauda equina or spinal nerve roots in a supportive fashion. Although root enhancement has been reported in many cases of GBS/AIDP, its presence is variable, and its absence does not exclude the diagnosis. In WNV-associated neuropathies, MRI findings are frequently normal, and imaging is used primarily to exclude alternative structural or central nervous system pathology. In our patient, the lack of nerve root enhancement, together with symmetric weakness, sensory involvement, albuminocytologic dissociation, and demyelinating electrodiagnostic findings, supported an immune-mediated polyradiculoneuropathy rather than anterior horn cell involvement [14]. Nerve conduction studies are fundamental to confirming demyelination. Prolonged distal motor latencies reflect impaired distal conduction. Relative preservation of sural sensory responses compared with abnormal upper-limb sensory responses is a classic supportive pattern in AIDP [15]. Slowed motor conduction velocities, conduction block, and temporal dispersion are hallmark findings. Abnormalities on electrodiagnostic testing must be present in at least two nerves; early studies may be normal [16]. F-waves and H-reflexes may be prolonged or absent, reflecting proximal conduction block or root-level demyelination [17]. Serial studies are essential for distinguishing AIDP from axonal GBS variants, as criteria rely heavily on evolving parameters [18].

Differential diagnosis analysis

The patient’s rapidly progressive symmetric weakness, areflexia, sensory involvement, and albuminocytologic dissociation strongly support AIDP. The presence of WNV IgM and IgG antibodies indicates recent infection and supports WNV as a plausible postinfectious trigger. CSF demonstrated elevated protein without pleocytosis, arguing against WNV meningoencephalitis or AFM, which typically demonstrates lymphocytic pleocytosis.

WNV-associated AFM was unlikely given the symmetric weakness, prominent sensory involvement, lack of autonomic dysfunction, and normal spinal MRI without ventral horn or root enhancement. Acute motor axonal neuropathy (AMAN) and acute motor and sensory axonal neuropathy (AMSAN) variants were considered; however, slowed conduction velocities, conduction block, and demyelinating features on electrodiagnostic testing favored AIDP over an axonal subtype. While serial studies would have further characterized the evolution of demyelination versus axonal loss, the initial study was already strongly supportive of AIDP.

Miller Fisher syndrome was unlikely in the absence of ophthalmoplegia or predominant cranial nerve involvement. Central nervous system etiologies such as transverse myelitis were excluded by normal MRI, absence of a sensory level, and lack of upper motor neuron signs. Toxic-metabolic neuropathies, including those related to alcohol or thiamine deficiency, were inconsistent with the acute onset, CSF profile, and electrophysiologic evidence of demyelination. Overall, clinical, CSF, imaging, and electrophysiologic findings most strongly support WNV-associated AIDP.

Pathophysiology

WNV-associated AIDP is thought to arise through immune-mediated mechanisms rather than direct viral invasion of peripheral nerves. Following infection, activation of innate and adaptive immune responses is essential for viral clearance; however, excessive or dysregulated immune activation may contribute to tissue injury. In severe WNV disease, elevated and atypically polarized T-cell responses have been reported, supporting a role for immune-mediated damage in disease pathogenesis [2]. The development of AIDP following WNV infection likely reflects mechanisms common to postinfectious immune-mediated neuropathies, including molecular mimicry, bystander T-cell activation, and dysregulated cytokine signaling [19]. In the context of WNV, alcohol use has been associated with increased risk of neuroinvasive disease, and broader autoimmune literature suggests that alcohol exerts dose-dependent immunomodulatory effects through cytokine regulation, NF-κB/NLRP3 signaling, and potential microbiome interactions; however, its specific role in postinfectious demyelinating neuropathy remains uncertain [20]. The potential influence of nicotine and cannabis on immune-mediated neurological disease is complex and incompletely understood. Experimental models of autoimmune demyelination demonstrate that nicotine can modulate inflammatory responses and reduce disease severity, but these data derive from central nervous system models and may not translate to peripheral demyelinating neuropathies [21]. Cannabis exerts complex and context-dependent immunomodulatory effects, with evidence demonstrating both immunosuppressive and proinflammatory influences depending on dose, duration, and biological context. However, current literature does not establish a causal relationship between cannabis use and autoimmune demyelinating disease, and cannabis has not been identified as a risk factor for West Nile neuroinvasive disease or WNV-associated AIDP. Therefore, any potential contribution of cannabis exposure in this patient should be considered theoretical and hypothesis-generating rather than evidence-based [5,22].

## Conclusions

This case highlights the diagnostic complexity of distinguishing WNV-associated AIDP from other neuroinvasive and immune-mediated neurological conditions. The patient’s rapid clinical progression, mixed demyelinating and axonal features on electrophysiology, and MRI findings notable for a nonspecific brain lesion without nerve root enhancement underscore the importance of early recognition and timely consideration of immunotherapy in appropriate clinical contexts, rather than attributing recovery to treatment alone. Substance exposure may modify immune responses, although its relevance in this setting remains speculative and hypothesis-generating. Ongoing investigation, including serial EMG/NCS to track demyelinating versus axonal evolution, CSF WNV IgM or PCR when available, and targeted antiganglioside antibody testing, may help further clarify the immunopathogenesis of WNV-associated AIDP and refine diagnostic confidence and management strategies in future cases.
